# Virus-Like Particles Derived From a Virulent Strain of Pest des Petits Ruminants Virus Elicit a More Vigorous Immune Response in Mice and Small Ruminants Than Those From a Vaccine Strain

**DOI:** 10.3389/fmicb.2020.00609

**Published:** 2020-04-23

**Authors:** Feihu Yan, Entao Li, Ling Li, Zachary Schiffman, Pei Huang, Shengnan Zhang, Guohua Li, Hongli Jin, Hualei Wang, Xinghai Zhang, Yuwei Gao, Na Feng, Yongkun Zhao, Chengyu Wang, Xianzhu Xia

**Affiliations:** ^1^Key Laboratory of Jilin Province for Zoonosis Prevention and Control, Changchun Veterinary Research Institute, Chinese Academy of Agricultural Sciences, Changchun, China; ^2^College of Veterinary Medicine, South China Agricultural University, Guangzhou, China; ^3^National Research Center for Exotic Animal Diseases, China Animal Health and Epidemiology Center, Qingdao, China; ^4^Department of Medical Microbiology and Infectious Diseases, University of Manitoba, Winnipeg, MB, Canada; ^5^National Microbiology Laboratory, Special Pathogens Program, Public Health Agency of Canada, Winnipeg, MB, Canada; ^6^College of Veterinary Medicine, Jilin Agricultural University, Changchun, China; ^7^College of Wildlife Resources, Northeast Forestry University, Harbin, China; ^8^College of Animal Science and Technology, Shihezi University, Shihezi, China; ^9^College of Veterinary Medicine, Jilin University, Changchun, China

**Keywords:** peste des petits ruminants virus, virus-like particles, small ruminants, virulent strain, vaccine strain, immune response

## Abstract

Peste des petits ruminants (PPRs) is highly contagious, acute or subacute disease of small ruminants caused by peste des petits ruminants virus (PPRV). To date, several studies have designed and evaluated PPRV-like particles (VLPs) as a vaccine candidate for the prevention and control of PPR, with the majority of these VLPs constructed using sequences derived from a PPRV vaccine strain due to its high immunogenicity. However, because of the lack of available genetic material and certain structural proteins and/or the alteration of posttranslational glycosylation modifications, the immunogenicity of VLPs derived from a vaccine strain is not always optimal. In this study, two PPRV VLP candidates, derived from either the lineage IV Tibet/30 virulent strain or the lineage II Nigeria 75/1 vaccine strain, were generated using a baculovirus system through the coexpression of the PPRV matrix (M), hemagglutinin (H), and fusion (F) proteins in the high expression level cell line High Five. These VLPs were then used to immunize mice, goats, and sheep followed by two boosts after primary immunization. Both VLPs were found to induce a potent humoral immune response as demonstrated by the high ratio of immunoglobulin G1 (IgG1) to IgG2a. In all animals, both VLPs induced high titers of virus-neutralizing antibodies (VNAs), as well as H- and F-specific antibodies, with the Tibet/30 VLPs yielding higher antibody titers by comparison to the Nigeria 75/1 VLPs. Studies in mice also demonstrated that the Tibet/30 VLPs induced a more robust interleukin 4 and interferon γ response than the Nigeria 75/1 VLPs. Goats and sheep immunized with both VLPs exhibited a robust humoral and cell-mediated immune response. Furthermore, our results demonstrated that the VLPs derived from the virulent lineage IV Tibet/30 strain were more immunogenic, inducing a more potent and robust humoral and cell-mediated immune response in vaccinated animals by comparison to the lineage II Nigeria 75/1 vaccine strain VLPs. In addition, VNA titers were significantly higher among animals vaccinated with the Tibet/30 VLPs by comparison to the Nigeria 75/1 VLPs. Taken together, these findings suggest that VLPs derived from the virulent lineage IV Tibet/30 strain are more immunogenic by comparison to those derived from the lineage II Nigeria 75/1 vaccine strain and thus represent a promising vaccine candidate for the control and eradication of PPR.

## Introduction

Peste des petits ruminants virus (PPRV), renamed to small ruminant morbillivirus in 2017 (referred to as PPRV throughout this study) (Amarasinghe et al., 2017), is the etiological agent of peste des petits ruminants (PPRs), a highly contagious and devastating transboundary disease, which affects nearly 30 million animals, mainly goats and sheep, annually across more than 70 countries worldwide. Peste des petits ruminants virus (genus *Morbillivirus*, family Paramyxoviridae) is a non-segmented, negative-sense RNA virus with a genome of ∼16 kb that encodes a total of six structural proteins [nucleocapsid (N), phosphoprotein (P), matrix (M), fusion (F), hemagglutinin (H), and polymerase (L)] and two non-structural proteins (V and C) (Gibbs et al., 1979; Bailey et al., 2005). Coexpression of the PPRV M, F, and H proteins results in the efficient assembly and release of virus-like particles (VLPs) (Wang et al., 2017). The M protein is the most abundant structural protein within the mature virion and plays a critical role in viral morphogenesis, acting as a driving force of virus budding (Haffar et al., 1999; Pohl et al., 2007). Proteins F and H are two surface expressed glycoproteins, which play an important role in attachment to the host cell, as well as mediating fusion of the viral envelope with the host cell membrane (Baron et al., 2016). While both the F and H proteins are potent inducers of a protective host immune response, the H protein is more immunogenic compared to F, ultimately stimulating a more robust humoral immune response, with the majority of virus-neutralizing antibodies (VNAs) being directed against the H protein (Sinnathamby et al., 2001; Renukaradhya et al., 2002; Rahman et al., 2003; Diallo et al., 2007).

Peste des petits ruminant is classified by the World Organization for Animal Health (OIE) as a notifiable terrestrial animal disease and is estimated to result in economic losses of US $1.4 million to $2.1 billion annually, mostly in Africa and Asia, mainly due to morbidity, mortality, production losses, and treatment costs. Furthermore, PPR also has severe negative impacts on food and job security, as well as livelihood, especially among women and children, exacerbating poverty and malnutrition, particularly among highly vulnerable rural communities (Kumar et al., 2014).

Following the successful global eradication of rinderpest in 2011, a global consensus was reached on the need to eradicate PPR (Taylor, 2016). In April 2015, a PPR global control and eradication strategy were endorsed during a conference in Côte d’Ivoire with the aim of eradicating PPR globally by 2030 (OIE and FAO, 2015). To implement this strategy, the Food and Agriculture Organization of the United Nations (FAO) and OIE, 2016 launched, in October 2016, an initial PPR global eradication program for 2017–2021. In accordance with this strategy, the Chinese government issued in December 2015 the National Eradication Program for PPR (2016–2020), with the goal of eradicating PPR countrywide by 2020 (Liu et al., 2018). Lessons learned from the global eradication of rinderpest in 2011 demonstrated that the use of a highly efficacious vaccine was critical to the campaign’s success, and as such, vaccination has been identified as the most suitable option for the control and eradication of PPR.

Sequence-based phylogenetic analysis has classified PPRV into four distinct lineages (I, II, III, and IV) (Forsyth and Barrett, 1995; Couacy-Hymann et al., 2002), but only one serotype exists. Lineages I, II, and III are most prominent among African and Middle Eastern countries, whereas lineage IV is most prominent among Asian countries (Wu et al., 2016) and more recently several African countries previously reporting only a single lineage (Kwiatek et al., 2011; Munir, 2015). Although there is only one serotype of PPRV, there are quantitative and qualitative differences in immune responses among different lineages (Hodgson et al., 2018). While PPRV VLPs derived from lineage II vaccine strains have proven promising as a differentiating infected from vaccinated animals (DIVA) vaccine candidate, the immunogenicity of VLPs derived from virulent strains still remains largely unknown (Li et al., 2014; Liu et al., 2015; Wang et al., 2017; Yan et al., 2019).

To this effect, we constructed two VLP vaccine candidates derived from the lineage IV Tibet/30 virulent strain and lineage II–attenuated Nigeria 75/1 vaccine strain respectively using a baculovirus system for the simultaneous coexpression of the codon-optimized M, F, and H proteins in insect cells. These VLPs were subsequently used to immunize mice, goats, and sheep, and the results revealed that the Tibet/30 VLPs were highly immunogenic, eliciting a more potent humoral and cell-mediated immune response among vaccinated animals by comparison to the Nigeria 75/1 VLPs and thus represents a prospective candidate vaccine for the control and eradication of PPR.

## Materials and Methods

### Cells and Viruses

Adherent *Spodoptera frugiperda* (Sf9) insect cells used for baculovirus rescue and propagation were maintained in Grace’s Insect Medium (Life Technologies, San Diego, CA, United States) and cultured at 27°C. High Five insect cells (BTI-TN-5B1-4) used for VLP production were grown in suspension in Express Five serum-free media (Thermo Fisher Scientific, Saint Louis, MO, United States) and cultured at 27°C on a temperate orbital shaker at 200 rpm. Propagation and titration of PPRV were done on African green monkey kidney cells (Vero), which were cultured in Dulbecco modified Eagle medium supplemented with 10% heat inactivated fetal bovine serum at 37°C with 5% CO_2_. Peste des petits ruminants virus vaccine strain Nigeria 75/1 was stored in our laboratory.

### Construction of Bacmid Transfer Plasmid

**FIGURE 1:**
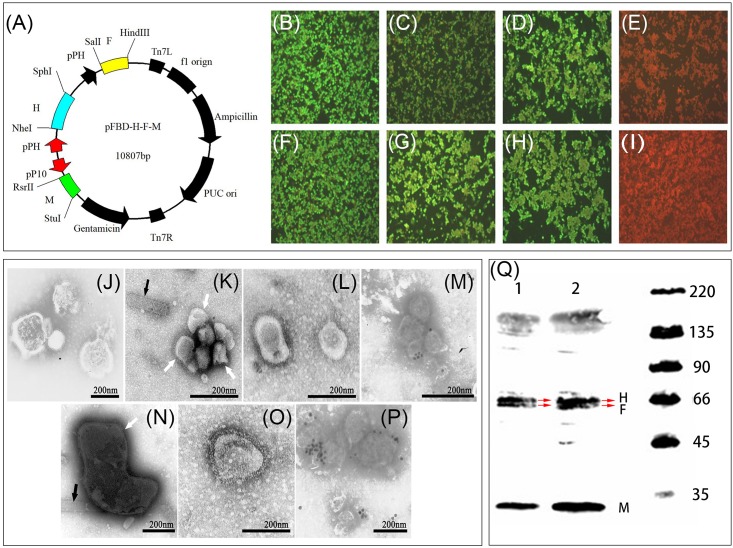
Generation and characterization of PPRV VLPs. **(A)** Schematic diagram for the recombinant plasmids pFBD-M-F-H. **(B–I)** Detection of the expression of M, F, and H. Sf9 cells were mock-infected or infected with rBVs rpFBD-M-F-H or wild-type baculovirus. Expression was evaluated by IFA using mouse anti-PPRV M, F, and H polyclonal antibody, respectively. Nigeria 75/1 M protein **(B)**, Nigeria 75/1 F protein **(C)**, Nigeria 75/1 H protein **(D)**, Tibet/30 M protein **(E)**, Tibet/30 F protein **(F)**, Tibet/30 H protein **(G)**, wild-type baculovirus-infected control **(H)**, and mock-infected control **(I)**. **(J–P)** Transmission electron microscopy images of virus and VLP preparations. Native PPRV particles **(J)**, residual baculoviruses (indicated by black triangles) in preparations of Nigeria 75/1 VLPs (indicated by white arrow) **(K)**, purified Nigeria 75/1 PPRV VLPs **(L)**, and immunogold-labeled Nigeria 75/1 VLPs stained with mouse anti-PPRV H polyclonal antibody followed by gold-labeled goat anti-mouse IgG antibody **(M)**. Residual baculoviruses (indicated by black triangles) in preparations of Tibet/30 VLPs (indicated by white arrow) **(N)**, purified Tibet/30 PPRV VLPs **(O)**, and immunogold-labeled Tibet/30 VLPs stained with mouse anti-PPRV H polyclonal antibody followed by gold-labeled goat anti-mouse IgG antibody **(P)**. **(Q)** Western blot depicting (1) Nigeria 75/1 and (2) Tibet/30 VLP protein expression using sheep polyclonal antibody. However, M, F, and H proteins incorporation in both VLPs were confirmed by Western blot analysis, which demonstrated both M (38 kDa), F (59 kDa), and H (68 kDa) consistent with their respective molecular sizes.

Codon optimized open reading frames for the PPRV M, F, and H genes from the PPRV virulent strain China/Tibet/Geg/07-30 (GenBank FJ905304.1) and vaccine strain Nigeria 75/1 (GenBank HQ197753.1) with restriction enzyme sequences (Table 1) were synthesized by Sangon Biotech (Shanghai, China) Co., Ltd. The synthetic codon optimized genes were cloned into Puc57-Simple plasmid, respectively. The M gene was inserted into a modified pFastBacDual vector under a p10 promoter flanked by *Rsr*II and *Stu*I restriction sites yielding the recombinant plasmid pFBD-M. Then the H gene was cloned into pFBD-M under a polyhedrin (pH) promoter digested with *Sal*I and *Hin*gIII to yield the recombinant plasmid pFBD-M-H. Subsequently, the F gene was cloned into the recombinant plasmid pFBD-M-H vector under the second pH promoter digested with *Nhe*I and *Sph*I yielding the recombinant transfer plasmid pFBD-M-F-H (Figure 1A). Bacmid transfer plasmids were transformed into *Escherichia coli* DH10TMBac competent cells (Life Technologies, United States) containing the AcMNPV baculovirus genome to obtain recombinant bacmids containing M, F, and H genes of the PPRV Tibet/30 and Nigeria 75/1 strains, respectively. Recombinant bacmids were identified by polymerase chain reaction using three pairs of gene specific primers for the two PPRV strains. The sequences of all primers used in this study are summarized in Table 1.

**TABLE 1:** Sequences of primers used in the present study.

Primer	Sequence (5′–3′)	Restriction enzyme site
M_Tibet_F	ACACCGGTCCGATGACCGAAATCTACGACTT	*Rsr*II
M_Tibet_R	ACACAGGCCTTTAGAGAATTTTAAAGAGGCC	*Stu*I
F_Tibet_F	ACACGTCGACATGACCAGGGTTGCTATC	*Sal*I
F_Tibet_R	ACACAAGCTTTTACAGAGAGCGAACGTAAG	*Hin*gIII
H_Tibet_F	ACACGCTAGCATGTCCGCTCAAAGGGAGAG	*Nhe*I
H_Tibet_R	ACACGCATGCTTACACGGGATTGCAAGTGAC	*Sph*I
M_Nigeria_F	ACACGGTCCGATGACCGAGATCTACGAT	*Rsr*II
M_Nigeria_R	ATACAGGCCTTTACAGGATCTTGAACAG	*Stu*I
F_Nigeria_F	ACAGTCGACATGACACGGGTCGCAACC	*Sal*I
F_Nigeria_R	ATACAAGCTTCTACAGTGATCTCACGTA	*Hin*gIII
H_Nigeria_F	ACAGCTAGCATGTCCGCACAAAGGGAA	*Nhe*I
H_Nigeria_R	ATACGCATGCTCAGACTGGATTACATGT	*Sph*I

*^*a*^Restriction enzyme sites are underlined.*

### Generation and Identification of Recombinant Baculoviruses

Recombinant baculoviruses (rBVs) expressing PPRV M, F, and H proteins from either the virulent Tibet/30 strain or vaccine Nigeria 75/1 strain were rescued using the Cellfectin II Reagent (Life Technologies, United States) as previously described (Yan et al., 2019). Briefly, Sf9 cells were seeded 24 h prior to transfection in a 6-well plate at a density of 8 × 10^5^ cells/well to achieve a confluency of 80–90%. For transfection, 3 μg of recombinant bacmid DNA diluted in 100 μL unsupplemented Grace’s Insect Medium was combined with 100 μL diluted Cellfectin II Reagent (8 μL Cellfectin II Reagent diluted in unsupplemented Grace’s Medium) vortexed briefly and subsequently incubated at room temperature for 15–30 min. The DNA–lipid mixture was then added onto the Sf9 cells and incubated at 27°C. After 72 h, first-generation rBVs (P1) were harvested and subsequently passaged for several generations in order to obtain high-titer baculovirus.

To confirm expression of the M, F, and H proteins, both rBVs were subjected to immunofluorescence assay (IFA) and Western blot (WB) as previously described (Yan et al., 2019). Briefly, Sf9 cells were infected with the two rBVs, as well as wild-type baculovirus at a multiplicity of infection (MOI) of 1. Fourth-eight hours after infection, the cells were either fixed with 80% acetone and subjected to IFA analysis or lysed in sodium dodecyl sulfate–polyacrylamide gel electrophoresis sample buffer followed by WB analysis.

### Generation and Purification of PPRV VLPs

To generate PPRV VLPs, suspension High Five cells were infected with the rBVs at an MOI of 5 and the supernatants harvested at 120 h after infection and subjected to centrifugation at 7,000 rpm for 30 min to pellet cells and debris. The resulting clarified supernatant was then subjected to ultracentrifugation at a speed of 30,000 rpm for 1.5 h at 4°C to pellet the VLPs. The VLPs were then purified by ultracentrifugation at 35,000 rpm for 1.5 h at 4°C using a sucrose density gradient of 20–40–60% (wt/vol) prepared in phosphate-buffered saline (PBS). Protein bands between 20 and 40% corresponding to the purified VLPs were harvested and subsequently subjected to ultracentrifugation at 35,000 rpm for 1.5 h at 4°C to remove the sucrose. Lastly, the concentration of the VLPs was quantified using the BCA protein assay kit as per the manufacturer’s protocol (Beyotime, Nanjing, China).

### Electron and Immunoelectron Microscopy

The presence and morphology of PPRV VLPs were evaluated by negative staining electron microscopy as previously described (Qi et al., 2015). For immunoelectron microscopy, PPRV VLPs were applied to a copper–rhodium (Cu-Rh) grid and incubated at room temperature for 60 min followed by incubation with sheep anti-PPRV antibodies (1:200 dilution) at 37°C for 60 min. The copper mesh was then incubated with a 1:50 diluted 10 nm gold-labeled mouse anti-sheep immunoglobulin G (IgG) antibody at 37°C for 30 min (Abcam, Cambridge, MA, United States), and the copper mesh subsequently observed under a transmission electron microscope.

### Immunization of Mice, Goats, and Sheep and Sera Collection

All live animal work was performed in accordance with guidelines from the Animal Welfare and Ethics Committee of the Changchun Veterinary Research Institute (permit no. SCXK-2012-017). The environment and housing facilities satisfied the National Standards of Laboratory Animal Requirements (GB 14925-2001) of China.

Eight-week-old female BALB/c mice were purchased from the Changchun Institute of Biological Products Co., Ltd., China, and randomly separated into three groups of 10. Mice were vaccinated intramuscularly in the gastrocnemius muscle with 50 μg PPRV Tibet/30 or Nigeria 75/1 VLPs in 50 μL PBS mixed with 50 μL AddaVax adjuvant. Mice in the control group were given 50 μL PBS mixed with 50 μL AddaVax adjuvant. All groups received a second and third booster immunization at 2 and 4 weeks following the primary immunization. For mice, whole blood was collected 2, 4, 6, and 8 weeks following primary immunization, and the sera subsequently separated and stored at −80°C for further analysis.

Nine outbred goats (12–24 months old) and nine outbred sheep (8–16 months old) were fed by the Zhaoyuan Gaojiatan Goat Farm (Shandong, China) and independently randomized into three groups of three animals each. Goats were multipoint vaccinated subcutaneously (s.c.) in the neck skin with 300 μg PPRV Tibet/30 or Nigeria 75/1 VLPs in 500 μμL PBS mixed with 1 mL AddaVax adjuvant. Goats in the control group were given 500 μL PBS mixed with 1 mL AddaVax adjuvant. All groups received a second and third booster immunization at three and 6 weeks following the primary immunization. Sheep were immunized using the same exact approach as for goats. For both goats and sheep, whole blood was collected through the jugular vein at 3, 6, 9, 12, and 15 weeks after primary immunization using 10 mL vacuum blood collection tubes and the serum subsequently separated and stored at −80°C for further analysis.

### Virus Neutralization Assay

Serum samples from mice, goats, and sheep were analyzed for PPRV-specific VNA titers using a microneutralization assay. Briefly, 50 μL 2-fold serial diluted inactivated sera were combined with 50 μL PPRV Nigeria 75/1 (100TCID_50_) in 96-well cell culture plate and incubated at 37°C and 5% CO_2_ for 1 h. Virus-only control wells and uninfected-cell control wells were included. Following incubation, 100 μL (2 × 10^5^ cells) of Vero cell suspension was added to each well, and cytopathic effect (CPE) observed 8 days after infection. Virus neutralizing antibody titers were defined as the highest serum dilution at which the CPE was inhibited by at least 50% (Yan et al., 2019).

### Enzyme-Linked Immuneospot Assays **for Cytokine Production in Mice**

Mouse splenocytes were harvested 2 weeks following the second immunization and stimulated with inactivated PPRV Nigeria 75/1 (10 μμg/mL). Cells producing interleukin 2 (IL-2), IL-4, IL-10, or interferon γ (IFN-γ) were identified using enzyme-linked immunospot assay (ELISpot) kits (Mabtech AB, Stockholm, Sweden; R&D Systems, Minneapolis, MN, United States) according to the manufacturer’s instructions. Spot-forming cells (SFCs) were counted using an automated ELISpot reader (AID ELISPOT reader-iSpot, AID GmbH, GER).

### Enzyme-Linked Immunosorbent Assay for PPRV-Specific Antibody and Cytokine

Mouse sera obtained 2 weeks following the third immunization were analyzed for PPRV-specific IgG, IgG1, and IgG2a. Antibody titers were measured using an indirect enzyme-linked immunosorbent assay (ELISA), as previously described (Yan et al., 2019). Purified inactive PPRV (Nigeria 75/1) was used as the coating antigen at a concentration of 2 mg/mL, and the optical density value was recorded at 450 nm absorbance.

To determine antibody responses to the F and H protein of PPRV, an indirect ELISA was developed using purified F and H proteins produced in house from either the virulent Tibet/30 strain or Nigeria 75/1 vaccine strain. Briefly, 96-well flat-bottomed plates were coated with purified F and H proteins (0.5 μg/well) overnight at 4°C and all subsequent steps performed as previously described (Yan et al., 2019). Total IgG was detected using a similar method: 96-well plates were coated with 0.05 μg purified inactivated PPRV (Nigeria 75/1), and horseradish peroxidase (HRP)–conjugated rabbit anti-goat IgG diluted 1:10,000 in PBST (Thermo Fisher Scientific, United States) used as the secondary antibody.

Cytokine responses in goats and sheep were evaluated using commercially available ELISA kits for goat or sheep IL-2, IL-4, IL-10, and IFN-γ (Cusabio, Burlington, NC, United States). The reagents, samples, and standards were prepared according to the manufacturer’s protocol.

### Data Analysis

Figures were generated using GraphPad Prism 8.0 software (GraphPad Company, SanDiego, CA, United States). Differences between means were evaluated using the one-way analysis of variance (ANOVA) or two-way ANOVA and were deemed significant at *P* ≤ 0.05.

## Results

### Generation and Identification of PPRV VLPs

The M, F, and H genes derived from either the PPRV virulent Tibet/30 or Nigeria 75/1 vaccine strain were cloned into a modified pFastBacDual plasmid, which could carry three exogenous genes under the control of a p10 and two pH promoters, respectively, as shown in Figure 1A. Recombinant bacmid was obtained after homologous reorganization in competent DH10bac cells, and rBVs were rescued in Sf9 insect cells following bacmid transfection. Subsequent infection of High Five insect cells with the two rBVs yielded PPRV VLPs derived from the virulent Tibet/30 strain and Nigeria 75/1 vaccine strain, respectively. Expression of the PPRV M, F, and H proteins was confirmed by IFA and WB (Figures 1B–I,Q). Furthermore, transmission electron microscopy revealed that the morphology of the VLPs resembled that of authentic PPRV containing spikes on the particle surface (Figures 1J,K,N). In addition, removal of residual baculovirus following purification by ultracentrifugation using a sucrose density gradient was confirmed by transmission electron microscopy (Figures 1L,O). Lastly, immunoelectron microscopy suggested that the two major PPRV immunogenic glycoproteins F and H, respectively, were incorporated into the VLPs (Figures 1M,P) and confirmed by WB (Figure 1Q).

### Characterization of the Humoral Immune Response to PPRV VLPs in Mice

**FIGURE 2:**
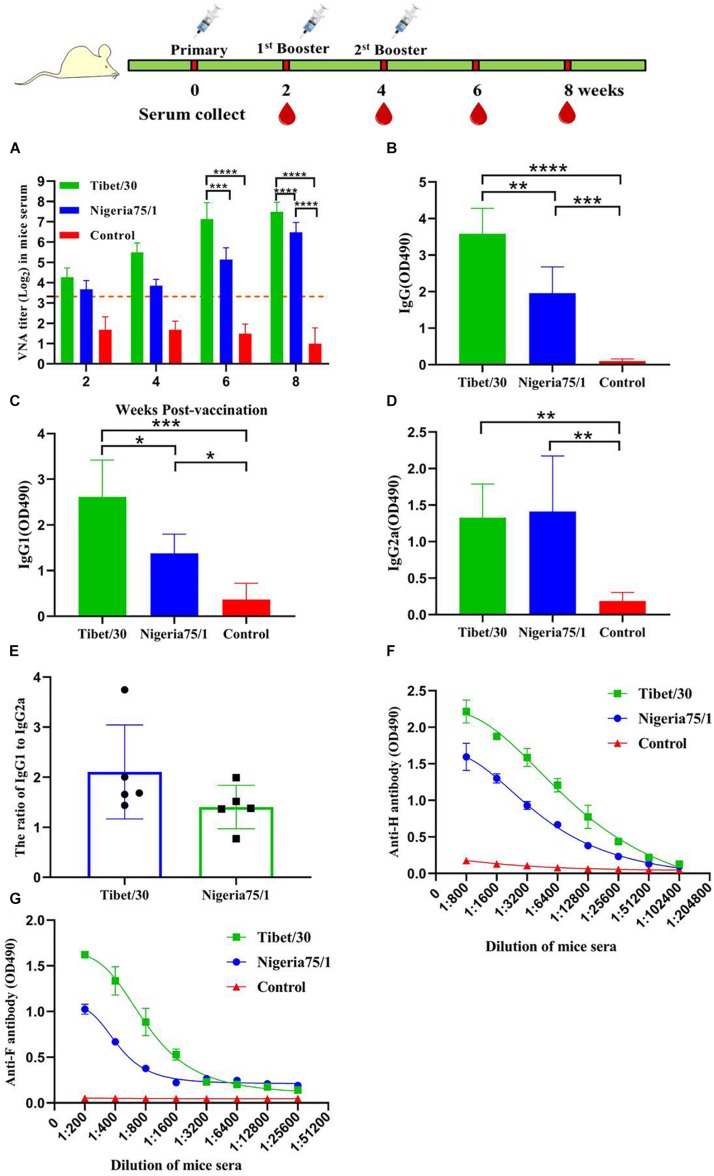
Virus-like particle immunization induces humoral immune response in mice. Mice were immunized thrice via s.c. route at 2 weeks’ interval with PPRV Tibet/30 VLPs, PPRV Nigeria 75/1 VLPs, or PBS, all with equal volume of adjuvant. Serum samples were collected 2, 4, 6, and 8 weeks after primary vaccination. **(A)** VNA titers were measured by virus neutralization assay. Dotted line represented antibody titers greater than 10, indicating positive serum conversion. **(B–D)** The specific anti-PPRV serum IgG and isotype responses were detected by ELISA. The serum dilution factor was 5,000. Serum IgG **(B)**, IgG1 **(C)**, and IgG2a **(D)**, responses were determined 6 weeks after the primary immunization. **(E)** The IgG1/IgG2a ratio was calculated. **(F,G)** Serum was collected from mouse 6 weeks after the primary immunization for analyzing F- and H-specific antibodies by ELISA. Data were depicted as the means ± SD for seven mice from each group and were analyzed by one- or two-way ANOVA (*P < 0.05, **P < 0.01, ***P < 0.001, ****P < 0.0001).

To evaluate the immunogenicity of the PPRV VLPs, mice were vaccinated with 50 μg PPRV Tibet/30 or Nigeria 75/1 VLPs and boosted 2 and 4 weeks after primary vaccination, after which VNA titers were determined using a microneutralization assay to assess the humoral immune response. At 2 weeks after primary vaccination, VNA titers from both the PPRV Tibet/30 and Nigeria 75/1 VLP-vaccinated groups exceeded 10 (Figure 2A), the standard minimum value as defined by the OIE, required for protection in goats immunized with live-attenuated PPRV Nigeria 75/1 (OIE, 2008; Yan et al., 2019). In the case of the Tibet/30 VLP-immunized group, VNA titers continued to increase to 2^5^–2^6^ and 2^7^–2^8^ at 4 and 6 weeks after primary vaccination, respectively, and those levels sustained at 2^7^–2^8^ at 8 weeks after primary vaccination (Figure 2A). By comparison, VNA titers from the Nigeria 75/1 VLP-immunized group increased to 2^3^–2^4^ and 2^5^–2^6^ four and 6 weeks after primary vaccination respectively and those levels sustained at 2^6^–2^7^ 8 weeks after primary vaccination (Figure 2A). In short, both PPRV VLPs elicited a potent humoral immune response in mice, resulting in the production of high amounts of VNAs, however, VNA titers were significantly higher at 6 and 8 weeks after vaccination in mice vaccinated with Tibet/30 VLPs by comparison to Nigeria 75/1 VLPs (Figure 2A).

To further evaluate the humoral immune responses induced by the two PPRV VLPs, antigen-specific total IgG and IgG1 and IgG2a titers were determined by ELISA. While both the Tibet/30 and Nigeria 75/1 VLPs induced the production of PPRV-specific IgG in mice, the Tibet/30 VLPs induced IgG titers significantly higher than Nigeria 75/1 VLPs (Figure 2B). Furthermore, both the PPRV Tibet/30 and Nigeria 75/1 VLP-vaccinated groups exhibited total IgG titers significantly higher than the PBS control group (Figure 2B). In the case of IgG1, the trend was similar to that observed for total IgG (Figure 2C). Both VLPs significantly upregulated IgG2a production; however, there was no significant difference among Tibet/30 and Nigeria 75/1 VLP-vaccinated groups (Figure 2D). The relatively high ratio of IgG1 to IgG2a for both the Tibet/30 and Nigeria 75/1 VLP-vaccinated mice suggested the activation of T_H_2-type immune response (Figure 2E).

Lastly, antibodies against the two major PPRV surface glycoproteins namely H and F were measured using an established ELISA. As shown in Figures 2F,G, both PPRV VLPs induced high levels of antibodies toward the H and F proteins, with the Tibet/30 VLPs inducing a more potent response by comparison to Nigeria 75/1 VLPs, which is consistent with the results obtained from the VNA assay. Taken together, these data demonstrate that the PPRV Tibet/30 VLPs elicited a more significant humoral immune response in mice by comparison to the PPRV Nigeria 75/1 VLPs.

### Characterization of the Cell-Mediated Immune Response in Mice Vaccinated With PPRV VLPs

**FIGURE 3:**
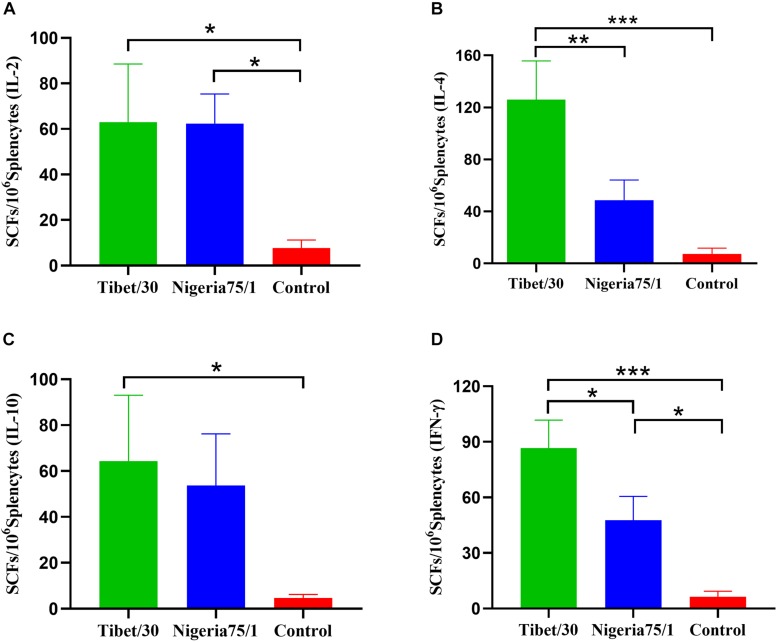
Virus-like particle immunization induces cell-mediated immune response in mice. Splenocytes from mice were stimulated with inactivated PPRV 2 weeks after the third immunization. Splenocytes producing IL-2 **(A)**, IL-4 **(B)**, IL-10 **(C)**, or IFN-γ **(D)** were identified by ELISpot. Data are depicted as the means ± SD of SFCs per million splenocytes from three mice in each group and were analyzed by one-way ANOVA (*P < 0.05, **P < 0.01, ***P < 0.001).

To investigate whether PPRV VLPs could elicit a cell-mediated immune response in mice, the activation of PPRV-specific IL-2, IL-4, IL-10, and IFN-γ in splenocytes were evaluated by ELISpot assays. The results demonstrated that the Tibet/30 VLPs elicited the activation of mouse splenocytes with significantly more SFCs for IL-2, IL-4, IL-10, and IFN-γ by comparison to the PBS control group (Figure 3). Although the Nigeria 75/1 VLPs exhibited significantly higher numbers of IL-2 and IFN-γ SFCs compared to the PBS control group, no significant difference was observed for IL-4 and IL-10 (Figure 3). Furthermore, the Tibet/30 VLPs appeared to be superior at eliciting IL-4 and IFN-γ production with significantly more SFCs when compared to Nigeria 75/1 VLPs (Figures 3B,D).

### Characterization of the Humoral Immune Response to PPRV VLPs in Goats and Sheep

**FIGURE 4:**
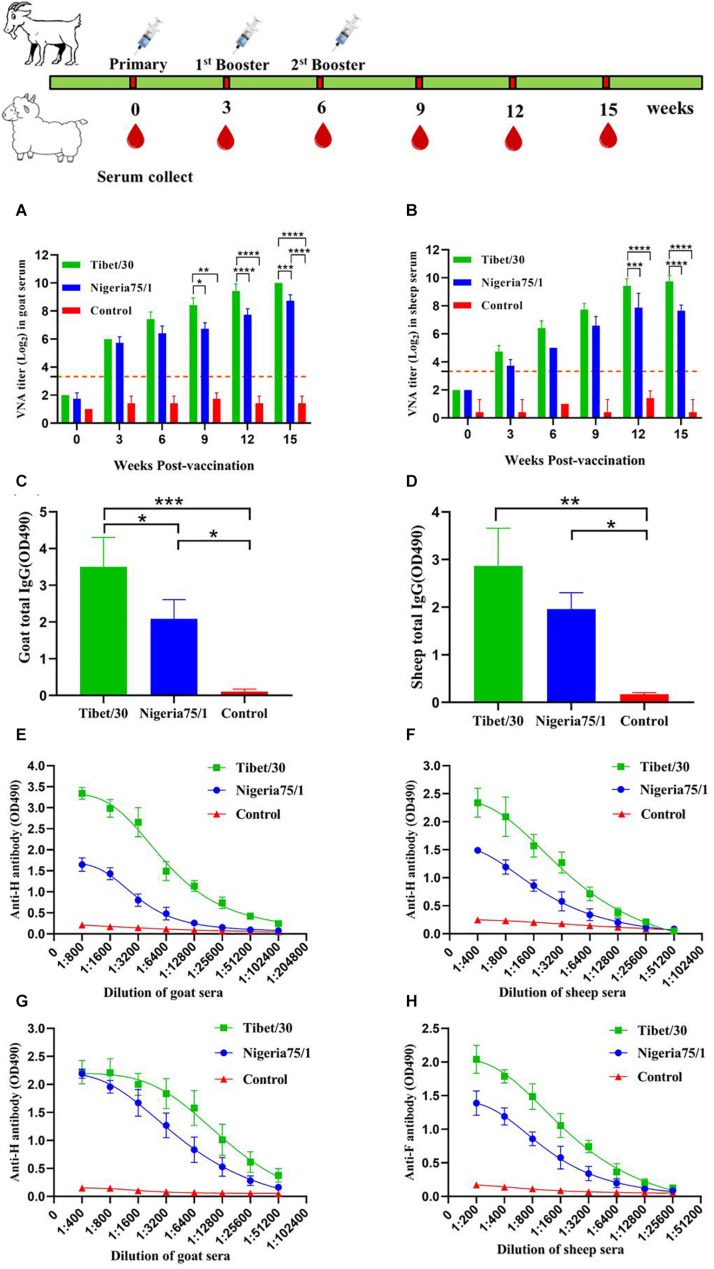
Virus-like particle immunization induces humoral response in goats and sheep. Goats or sheep were immunized thrice via s.c. route at 3 weeks’ interval with PPRV Tibet/30 VLPs, PPRV Nigeria 75/1 VLPs, or adjuvant. **(A,B)** VNA titers were measured at 3, 6, 9, 12, and 15 weeks after primary vaccination. Dotted lines represent antibody titers greater than 10, indicating positive serum conversion. **(C,D)** Total goat serum IgG **(C)** and total sheep serum IgG **(D)** responses were determined 9 weeks after the primary immunization. **(E–H)** Serum was collected from each goat or sheep 3 weeks after the second booster immunization for analyzing PPRV F- and H-specific antibodies by ELISA. Data are depicted as the means ± SD for three goats of sheep from each group and were analyzed by one-way or two-way ANOVA (*P < 0.05, **P < 0.01, ***P < 0.001, ****P < 0.0001).

Goats and sheep are among the two most susceptible hosts to PPRV infection and as such represent the ideal animal models for evaluating the immunogenicity and humoral immune response induced by PPRV VLPs. To evaluate the immunogenicity of the PPRV VLPs, both goats and sheep were vaccinated with 300 μg PPRV Tibet/30 or Nigeria 75/1 VLPs and boosted 3 and 6 weeks after primary vaccination, after which VNA titers were determined using a microneutralization assay to assess the humoral immune response. At 3 weeks after primary vaccination, VNA titers from both the PPRV Tibet/30 and Nigeria 75/1 VLP-vaccinated groups exceeded the OIE standard of 10 for both goats and sheep (Figures 4A,B). In goats, VNA titers gradually increased and reached statistical significance by comparison to the PBS control group at 9 weeks after primary vaccination, in the case of the Tibet/30 VLP-immunized group and remained statistically significant 12 and 15 weeks after primary vaccination (Figure 4A). In contrast, the Nigeria 75/1 VLP-immunized group only reached statistical significance by comparison to the PBS control group 15 weeks following primary vaccination (Figure 4A). In contrast, VNA titers of Tibet/30 VLP-vaccinated sheep only reached statistical significance by comparison to the PBS control group 12 weeks after primary vaccination with this significance sustained 15 weeks after primary vaccination (Figure 4B), whereas VNA titers for Nigeria 75/1 VLP-vaccinated sheep did not reach statistical significance by comparison to the PBS control at any point (Figure 4B). Furthermore, Tibet/30 VLP-immunized goats exhibited significantly higher VNA titers by comparison to Nigeria 75/1 VLP-immunized goats 9, 12, and 15 weeks after initial vaccination (Figure 4A), whereas in sheep this significance was only observed 12 and 15 weeks after initial vaccination (Figure 4B).

As in mice, the humoral immune responses in goats and sheep induced by the two PPRV VLPs were further evaluated by quantifying total antigen-specific IgG titers by ELISA. Although both the Tibet/30 and Nigeria 75/1 VLPs induced the production of PPRV-specific IgG in goats, the Tibet/30 VLPs induced IgG titers significantly higher than Nigeria 75/1 VLPs 9 weeks after initial vaccination (Figure 4C). In contrast, there was no statistical difference in total IgG among sheep vaccinated with either the Tibet/30 or Nigeria 75/1 PPRV VLPs, which may be attributed to the lower susceptibility of sheep to PPRV (Figure 4D). Additionally, in both goats and sheep, total IgG levels for both the PPRV Tibet/30 and Nigeria 75/1 VLP-vaccinated groups were significantly higher than the PBS control group (Figures 4C,D).

Furthermore, all animals developed high levels of PPRV-specific antibodies against the two major PPRV surface glycoproteins, namely, H and F, with the Tibet/30 VLPs yielding higher titers by comparison to Nigeria 75/1 VLPs in both goats and sheep (Figures 4E–H). In general, the humoral immune indicators evaluated suggest that sheep mounted a less robust humoral immune response than goats, which could possibly be attributed to their reduced susceptibility to PPRV infection. Overall, these data are in agreement with those observed in mice and suggest that although both PPRV VLPs elicited a strong humoral immune response in goats and sheep, the Tibet/30 VLPs were more effective at inducing this response by comparison to Nigeria 75/1 VLPs.

### Characterization of the Cell-Mediated Immune Response in Goats and Sheep Vaccinated With PPRV VLPs

**FIGURE 5:**
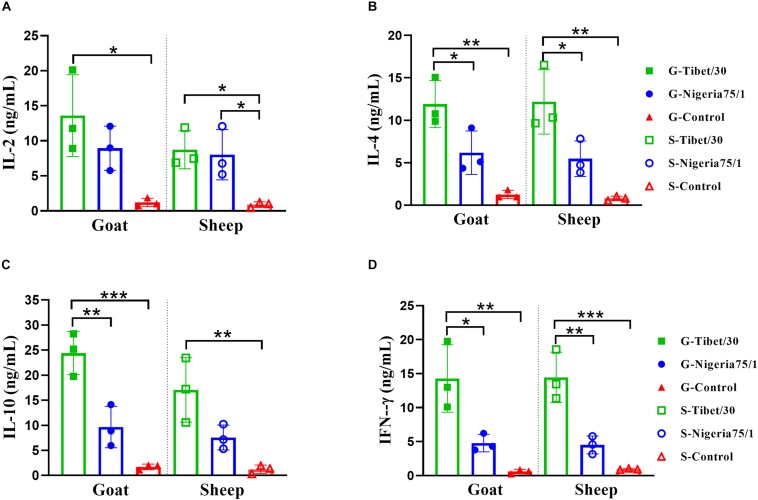
Virus-like particle immunization induces significant cytokine response in goats and sheep. **(A–D)** Serum was collected 3 weeks after the second immunization from animals immunized with PPRV Tibet/30 VLPs, PPRV Nigeria 75/1 VLPs, or adjuvant and were analyzed for the production of IL-2 **(A)**, IL-4 **(B)**, IL-10 **(C)**, and IFN-γ **(D)**. Data are depicted as the means ± SD for three goats or sheep from each group and were analyzed by one-way ANOVA (*P < 0.05, **P < 0.01, ***P < 0.001).

Cell-mediated immune responses were evaluated by quantifying the levels of secreted IL-2, IL-4, IL-10, and IFN-γ in the serum of PPRV VLP-vaccinated goats and sheep as well as control animals by ELISA. In both goats and sheep, all four cytokines were significantly elevated in animals vaccinated with the Tibet/30 VLPs by comparison to the PBS control group (Figure 5). Despite, both goats and sheep vaccinated with Nigeria 75/1 VLPs exhibiting elevated levels of all four cytokines, none with the exception of IL-2 in sheep, were significantly elevated by comparison to the PBS control group (Figure 5). None of the animals in either control group exhibited a pronounced cytokine response as expected (Figure 5). Furthermore, the levels of IL-4 and IFN-γ were significantly elevated in both goats and sheep vaccinated with Tibet/30 VLPs by comparison to Nigeria 75/1 VLPs (Figures 5B,D). In addition, IL-10 levels were significantly elevated in goats but not sheep vaccinated with Tibet/30 VLPs by comparison to Nigeria 75/1 VLPs (Figure 5C). In both animals, there was no statistical difference in the levels of IL-2 among Tibet/30 or Nigeria 75/1 VLP-vaccinated groups (Figure 5A). Taken together, these data indicate that both PPRV VLPs elicited a cell-mediated immune response in goats and sheep, with the Tibet/30 VLPs inducing an overall more pronounced response than Nigeria 75/1 VLPs.

## Discussion

The production of VLPs using a baculovirus/insect cell expression system has proven to be an efficient strategy for vaccine development. Several VLP-based vaccines have been licensed and commercialized, such as Engerix-B^®^, 2012 (GSK, March 2012) and Cervarix^®^, 2011 (GSK, July 2011) against hepatitis B virus and human papillomavirus, respectively, as well as the veterinary vaccine Porcilis^®^ (Kushnir et al., 2012) against porcine circovirus type 2. Most often, VLPs are constructed using sequences derived from a virus vaccine strain due to its well-established immunogenicity. However, because of the lack of genetic material and certain structural proteins and/or the alteration of posttranslational glycosylation modifications, the immunogenicity of VLPs derived from a vaccine strain may not always be optimal. In this study, we constructed two PPRV VLP vaccine candidates derived from the sequences of either the virulent Tibet/30 or attenuated Nigeria 75/1 PPRV strains using a baculovirus system for the simultaneous coexpression of the codon-optimized M, F, and H proteins in insect cells. These VLPs were subsequently used to immunize mice, goats, and sheep and their immunogenicity compared by evaluating the magnitude of the humoral and cell-mediated immune responses they induced. Animal experiments demonstrated that both PPRV VLPs were capable of eliciting humoral and cell-mediated immune response in mice, goats, and sheep, with the Tibet/30 VLPs exhibiting a greater immunogenicity by comparison to the Nigeria 75/1 VLPs. Together, these data suggest that both PPRV VLPs represent suitable vaccine candidates for the control and eradication of PPR, with the Tibet/30 VLPs being the most promising candidate because of its greater immunogenicity. This conclusion also provides a way to improve VLP immunogenicity through the use of sequences derived from a PPRV strain rather than a vaccine strain.

A number of studies have previously explored the formation of PPRV VLPs and have demonstrated that coexpression of the PPRV M and N proteins are sufficient for the production of spikeless PPRV VLPs in insect cells. However, because of the lack of the PPRV H and F surface glycoproteins, which are the most immunological relevant determinants, these VLPs were unable to elicit an efficient protective immune response (Liu et al., 2014). On the other hand, coexpression of the N, M, F, and H proteins resulted in the successful assembly and release of PPRV VLPs in either Vero (Wang et al., 2017) or insect cells (Yan et al., 2019) and was capable of inducing strong humoral and cell-mediated immune responses in mice and goats (Yan et al., 2019). Furthermore, it has been demonstrated that expression of the M protein alone is sufficient for the assembly and release of PPRV VLPs; however, in the absence of M, no PPRV VLPs were released for any combination of N, H, and F proteins (Wang et al., 2017). While the expression of the F protein alone could support low levels of VLP assembly, no release was observed in the absence of M, further highlighting its crucial role as a structural protein in the assembly and release of VLPs (Wang et al., 2017). In contrast to the F protein, expression of the N or H proteins alone could neither support assembly nor release of VLPs (Wang et al., 2017). In our study, we constructed PPRV VLPs through the coexpression of the M, F, and H proteins respectively, with the M protein acting as the structural protein supporting assembly and release of the VLPs and the two glycoproteins, namely, F and H acting as the main immunogenic determinants on the surface of the PPRV VLPs. We chose to omit the N protein because of its non-essential role in the assembly and release of PPRV VLPs, as well as the convenience of using commercial kits for DIVA. Through the coexpression of the M, F, and H proteins, we were successful in generating PPRV VLPs derived from both the virulent Tibet/30 and attenuated Nigeria 75/1 PPRV strains, using a baculovirus/insect cell expression system, with the morphology of these VLPs resembling authentic PPRV containing spikes protruding from the particulate surfaces (Figure 1A).

To simplify the production process and reduce baculovirus contamination during purification, we constructed a single rBV carrying three codon-optimized exogenous genes encoding the M, F, and H proteins, respectively. All three proteins were expressed under the control of p10 and two pH promoters, respectively (Figure 1). This approach of using a single rBV expressing all three viral proteins rather than three separate rBVs each expressing one viral protein reduces the amount of residual baculovirus within the harvested supernatant, thus benefiting subsequent purification.

Peste des petits ruminants virus–like particles display antigenic epitopes in the correct conformation and in a highly repetitive manner, leading to crosslinking of B-cell immunoglobulin receptors (Bachmann and Zinkernagel, 1997; Grgacic and Anderson, 2006; Kushnir et al., 2012). This reaction stimulates B-cell proliferation and upregulation of both MHC class II and costimulatory molecules that allow for subsequent interactions with T-helper cells, which trigger immunoglobulin secretion, affinity maturation, and the long-lived memory B cells (Chackerian, 2007). Our results revealed that both PPRV VLPs were capable of eliciting a humoral immune response in mice, goats, and sheep, which resulted in the production of VNAs to titers sufficient for protection against PPRV infection. These data suggest that both the virulent Tibet/30 and vaccine Nigeria 75/1 PPRV strains share common neutralizing epitopes; however, those on the Tibet/30 strain appear to be more immunogenic, ultimately eliciting a more robust humoral immune response. These findings are further supported by higher levels of H- and F-specific antibody titers among mice, goats, and sheep vaccinated with the Tibet/30 VLPs as compared to those vaccinated with the Nigeria 75/1 VLPs. Additionally, the relatively high IgG1/IgG2a ratio in mice indicated that both PPRV VLPs elicited a T_H_2-preferred immune response favoring humoral immunity and was more pronounced among Tibet/30 VLP-vaccinated animals compared to those vaccinated with the Nigeria 75/1 VLPs. Although these findings are in agreement with those observed by Li et al. (2014), they contradict our previous findings (Yan et al., 2019) and can possibly be attributed to differences in adjuvant and/or the lack of the N protein within the VLPs, which has been found to induce a CTL response (Mitra-Kaushik et al., 2001).

A number of cytokines were elevated, modulating an antiviral immune response upon antigen recognition and presentation. Analysis of ELISpot assays detecting IL-2–, IL-4–, IL-10–, and IFN-γ–secreting mouse splenocytes showed that the Tibet/30 VLP-vaccinated group exhibited significantly increased levels of all four cytokines as compared to the PBS control group. In contrast, mice vaccinated with the Nigeria 75/1 VLPs only demonstrated significantly increased levels of IL-2 and IFN-γ as compared to the PBS control group, with the levels of IL-4 and IL-10, although slightly upregulated not reaching statistical significance. A similar trend was observed in both goats and sheep whereby all four cytokines were significantly elevated in animals vaccinated with the Tibet/30 VLPs by comparison to the PBS control group. Although both goats and sheep vaccinated with Nigeria 75/1 VLPs had elevated levels of all four cytokines, none with the exception of IL-2 in sheep were significantly elevated by comparison to the PBS control group. Interleukin 4 produced by T_H_2 cells drives the maturation of B cells into plasma cells, resulting in antibody production, isotype switching, and affinity maturation (Fang et al., 2007). Interleukin 10, which is also secreted by T_H_2 cells, inhibits the activation of T_H_1 cells and ultimately their production of cytokines (Moore et al., 2001). Interestingly, IL-4 and IFN-γ levels were significantly elevated in mice, goats, and sheep vaccinated with Tibet/30 VLPs by comparison to Nigeria 75/1 VLPs. In addition, IL-10 levels were significantly elevated in goats but not sheep vaccinated with Tibet/30 VLPs by comparison to Nigeria 75/1 VLPs. Taken together, these data support the conclusion that the PPRV VLPs elicited a T_H_2-preferred immune response favoring humoral immunity, which was more pronounced among Tibet/30 VLP-vaccinated animals compared with those vaccinated with Nigeria 75/1 VLPs. Overall the immune indicators in this study were more or less higher than those observed in our previous study and is likely due to the use of AddaVax as the adjuvant rather than Freund’s complete adjuvant (Yan et al., 2019). AddaVax is a squalene-based oil-in-water nanoemulsion based on the formulation of MF95 that has been demonstrated to elicit both cellular and humoral immune responses and licensed in Europe for adjuvanted flu vaccines (Vesikari et al., 2011; Alving et al., 2012; Calabro et al., 2013).

**FIGURE 6:**
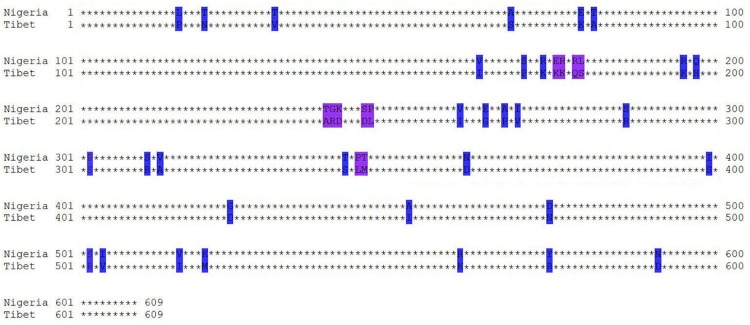
Amino acid sequence alignment analyses of H protein. Homology of the PPRV H protein from PPRV Tibet/30 and Nigeria75/1 strains was analyzed using MEGA (Molecular Evolutionary Genetics Analysis) version 6.0. Capital letters represent the location of amino acid site mutations, and * represents the amino acid without difference between two strains.

A recent study by Hodgson et al. (2018) reported qualitative and quantitative differences in immunogenicity among PPRV lineages. To this effect, we set out to compare the immunogenicity of PPRV VLPs derived from PPRV strains of different lineages and virulence, namely, the lineage IV Tibet/30 virulent strain and the lineage II–attenuated Nigeria 75/1 vaccine strain. The PPRV lineage II Nigeria 75/1 strain was first isolated from a goat that succumbed to PPRV infection and later passaged on Vero cells to yield the first PPR-attenuated vaccine (Taylor and Abegunde, 1979; Diallo et al., 1989). The PPRV lineage IV Tibet/30 strain was isolated from a sick goat in July 2007 during the first outbreak of PPR in southwestern Tibet of China (Wang et al., 2009; Wu et al., 2016). The attenuated Nigeria 75/1 vaccine strain (lineage II) and virulent Tibet/30 strain (lineage IV) share 97, 96.5, and 92.9% similarity at the amino acid level for the M, F, and H proteins, respectively. The H protein of morbilliviruses is highly immunogenic and has been found to be a major inducer of both humoral and cell-mediated immune responses (Berhe et al., 2003). As such, the low homology in H proteins among the two PPRV strains under study could possibly account for the differences in immunogenicity observed for the two PPRV VLPs. The H protein of PPRV is a 609-residue type II integral membrane glycoprotein (Yu et al., 2017). The two PPRV strains under investigation differ by a total of 43 amino acids distributed among the N-terminal cytoplasmic tail (three mutants), stalk region (three mutants), and C-terminal globular head (37 mutants) containing the receptor binding site and immune epitopes, respectively (Figure 6; Colf et al., 2007; Yu et al., 2017). No mutants were observed in the transmembrane region of the PPRV H protein. B-cell epitope regions have been mapped to two discontinuous regions on the PPRV H protein from aa263-368 and aa538-609 (Renukaradhya et al., 2002). Interestingly, 11 mutants (25.6%) are located within aa263-368 and could possibly account for the differences in humoral immune responses observed among Tibet/30 and Nigeria 75/1 VLP-vaccinated animals. Frequent differences were also observed at aa163-179, the initial portion of head region N-terminal domain, and may represent a novel antigenic determinant. Lastly, differences in amino acids among the PPRV M and F proteins could also contribute to the differences in immunogenicity observed between the two PPRV VLPs by influencing the amount of antigenic proteins incorporated onto VLP surface, as well as the attachment and uptake rate of VLPs to host cells, which requires further investigation.

## Data Availability Statement

All datasets generated for this study are included in the article/supplementary material.

## Ethics Statement

All live animal work was performed in accordance with guidelines from the Animal Welfare and Ethics Committee of the Changchun Veterinary Research Institute (Permit No. SCXK-2012-017). The environment and housing facilities satisfied the National Standards of Laboratory Animal Requirements (GB 14925-2001) of China.

## Author Contributions

FY, NF, CW, and YZ conceived the study and designed experiments. FY, EL, LL, and SZ performed the animal experiments. FY, EL, HW, GL, and NF performed *in vitro* experiments and analyzed data. FY, PH, HJ, and XZ interpreted the data. FY, EL, LL, and ZS wrote the manuscript. ZS, YG, and XX reviewed the manuscript.

## Conflict of Interest

The authors declare that the research was conducted in the absence of any commercial or financial relationships that could be construed as a potential conflict of interest.
